# Growth and Survival in Small Hepatocellular Carcinoma

**DOI:** 10.1155/1994/65956

**Published:** 1994

**Authors:** Kunio Okuda

**Affiliations:** First Department of Medicine Chiba University School of Medicine Chiba 260 Japan


2. Delius, R.E., Frankel, F. and Coran, A.G. (1989) A comparison between operative and non-operative management of blunt injuries to the liver and spleen in adult and pediatric patients. *Surgery*, **106**, 788--793
3. Hollands, M.J. and Little, J.M. (1991) Non-operative management of blunt liver injuries. *Brit.J.Surg.*, **78**, 968–972
4. Kahn, D., Hickman, R., Dent, D.M. and Terblanche, J. (1986) For how long can the liver tolerate ischaemia? *Eur.Surg.Res.*, **18**, 277–282
5. Schrock, T. and Blaisdell, F.W. (1968) Management of blunt trauma to the liver and hepatic veins. *Arch.Surg.*, **96**, 698–704
6. Hollands, M.J. and Little, J.M. (1990) Hepatic venous injury after blunt abdominal trauma. *Surgery*, **107**, 149–152
7. Ciresi, K.F. and Lim, R.C. Jr. (1990) Hepatic vein and retrohepatic vena caval injury. *World J.Surg.*, **14**, 472–477
8. Krige, J.E.J., Worthley, C.S. and Terblanche, J. (1990) Severe juxtahepatic venous injury: survival after prolonged hepatic isolation without shunting. *HPB Surgery*, **3**, 39–45

J. M. Little
Department of Surgery
Westmead Hospital
Westmead N.S.W. 2145
Australia

### GROWTH AND SURVIVAL IN SMALL HEPATOCELLULAR CARCINOMA

### ABSTRACT

*Barbara, L., Benzi, G., Gaiani, S., Fusconi, F., Zironi, G., Sirongo, S., Rigamonti, A., Barbara, C., Grigioni, W., Mazziotti, A., Bolondi, L. (1992) Natural history of small untreated hepatocellular carcinoma in cirrhosis: A multivariate analysis of prognostic factors of tumour growth rate and patient survival. Hepatology; 16: 132–137.*

**We analyzed the growth pattern of tumor masses and the survival of 39 asymptomatic Italian patients with a total of 59 small (≤ 5 cm in diameter) hepatocellular carcinomas arising from cirrhosis. The total length of the observation period ranged from 90 to 962 days, with an average of 364 ± 229 (mean ± S.D.). Doubling time ranged from 27.2 to 605.6 days (mean ± S.D., 204.2 ± 135; median = 171.6 days). Three different growth patterns were recognized: (a) tumors with no or very slow initial growth pattern (doubling time > 200 days), 10 cases (37%); (b) tumors with declining growth rate over time, 9 cases (33.4%); and (c) tumors with almost constant growth rate, 8 cases (29.6%). Using the stepwise discriminant analysis, we found a score based on albumin, alcohol intake, number of nodules, echo pattern and histological type that allowed a correct prediction of short doubling time (≤ 150 days) in 55.6%, medium doubling time (151 to 300 days) in 60% and long doubling time (> 300 days) in 100% of cases. The estimated survival rate of the 39 patients,**

**calculated by the Kaplan-Meier method was 81% at 1 yr, 55.7% at 2 yr and 21% at 3 yr. Stepwise discriminant analysis showed that a score based on sex, HBsAg status, alcohol consumption, ascites, γ-glutamyltranspeptidase, prothrombin time, Child-Pugh class and all the sonographical parameters could predict 2-yr survival in 100% of cases. We conclude that great variability of growth patterns exists among and within small hepatocellular carcinomas. Prediction of subsequent growth rate is unreliable in most cases. The sonographical characteristics, together with the histological features, can, however, help in identifying cases with long doubling time (> 300 days). The discriminant analysis on survival of cirrhotic patients with small hepatocellular carcinomas demonstrates that the underlying liver disease plays a key role in the long-term survival probability. (Hepatology, 1992; 16:132–137.)**

### PAPER DISCUSSION

KEY WORDS: Small hepatocellular carcinoma, hepatocellular carcinoma growth rate

With the recent progress in imaging diagnosis, small space occupying lesions have come to be detected in the liver[1], and ultrasonography can now detect an hepatic mass smaller than 2 cm[2]. In the past, diagnosis of hepatocellular carcinoma (HCC) was possible only in symptomatic patients and the tumor size was invariably larger than 5 cm on detection. The Japanese gastroenterologists are to be credited for developing the diagnostic strategy or practice for early detection of HCC, namely, patients with cirrhosis are followed at a short regular interval with ultrasound examination and serum alpha-fetoprotein (AFP) measurement[3]. The same practice was immediately adopted in Taiwan, and later by the north Italian colleagues, notably Luigi Bolondi and Massimo Colombo. It is of interest in this connection that the incidence rate for HCC has been rising both in Italy[4] and Japan[5], the increase being accounted for by chronic hepatitis C virus infection[6,7].

When one detects a small HCC in a patient with cirrhosis, what should be done? Currently, a number of therapeutic modalities for the treatment of HCC are available, such as hepatic resection, orthotopic liver transplantation, chemotherapy, arterial embolization, lipiodolization, intratumor ethanol injection, irradiation, immunotherapy, etc. The prognosis of such small HCC depends on the speed of tumor growth as well as on the severity of cirrhosis. For the selection of therapeutic modality, therefore, one has to take into consideration the degree and speed of cirrhotic changes and growth rate of the HCC in each patient; the natural history of the disease was almost totally unpredictable in the past.

In this study, Barbara, Bolondi and their group in Bologna attempted to determine how well one can predict the speed of tumor growth and survival from the clinical and biochemical data at the time of tumor detection. Their study was based on a retrospective analysis of 39 asymptomatic Italian patients with a total of 59 small HCC lesions (less than 5 cm) associated with cirrhosis. They followed them for an average of 364 days while measuring the tumor doubling time (DT) without giving any specific treatment. They carried out the stepwise discriminant analysis with the data on albumin, bilirubin, prothrombin time, gamma-glutamyltranspeptidase (GGT), HBsAg, ferritin, AFP, excessive alcohol intake, ascites,

Child-Pugh class, number of tumor nodules, echo characteristics, tumor boundaries, histological type and Edmondson-Steiner's grade of differentiation[8]. They found that albumin, alcohol, number of nodules, echo pattern and histological type allowed a correct prediction of very long DT, and that survival at 2 years could also be predicted from the score calculated from sex, HBsAg, alcohol, ascites, GGT, prothrombin time, Child-Pugh class and sonographic parameters. It was in 1986 that Ebara *et al.*[9] first suggested that sonographic charcteristics were correlated with tumor growth speed, and this study seems to corroborate these observations. However, they emphasize that prediction of DT is generally difficult, and that the underlying liver disease plays a key role in the long term survival probability. Earlier, Calvet *et al.*[10] in Spain carried out a multivariate analysis based on clinical parameters that included toxic syndrome and metastases in 206 patients, but these patients were mostly advanced cases. The Spanish study and the current one are not comparable, and the latter is applicable only to small HCC.

In this study, varying growth rates were grouped into short (less than 150 days), average (150–300 d) and very long (more than 300 d) DT. However, the growth rate changed suddenly to a faster or a slower rate in a number of cases; a separate analysis may be required for such cases. In calculating the risk factor, such variables as albumin, bilirubin, GGT, AFP and prothrombin time were put into dichotomous variables; present = 1, absent = 0. They should have been dichotomized above and below a certain value, or at least more information is needed for the reader interested in using the proposed method for assessing the prognosis.

In the discussion, they point out certain differences between north Italian and Asian patients; tumor grows slower and patients live longer without treatment in north Italy. Although they comment on differences in the predictive value of AFP between their study and two reports from Asia[11,12], these studies were based on different sorts of patients and different modes of AFP value analysis, and this reviewer does not see any basic difference. A one-point value of AFP in a small HCC itself is of little prognostic value as the authors emphasize, but for more advanced HCC, AFP at diagnosis may be of prognostic significance[12]. They failed to show correlation between severity of cirrhosis and DT; a small HCC found in a liver with advanced cirrhosis tends to grow slowly in Japan, although this tendency has not been statistically documented. It is now established that malignant transformation is a multistep process, and the molecular events that underlie this process vary with the carcinogenic factors[7]. It is no surprise that the natural history differs somewhat between Italy and the Far East. Despite these geographic differences suggested by this study, their findings are of practical relevance regardless of the global region for those who are engaged in the diagnosis and treatment of HCC patients. The study was well planned and executed, and the results provide an important piece of information on the prognosis of HCC. While realizing that to detect a small HCC in 39 patients in a European country really requires an enormous amount of work and time, this reviewer suggests that they reanalyze these parameters in the future when they have accumulated a greater number of such patients.

### REFERENCES

1. Okuda, K. (1986) Early recognition of hepatocellular carcinoma. *Hepatology*, **6**, 729–738
2. Ohto, M., Ebara, M. and Okuda, K. (1987) Ultrasonography in the diagnosis of hepatic tumor. In: Okuda, K., Benhamou, J-P. eds. *Neoplasms of the liver*. Tokyo:Springer-Verlag, 251–258

3. Shinagawa, T., Ohto, M., Kimura, K., *et al.* (1984) Diagnosis and clinical features of small hepatocellular carcinoma with emphasis on the utility of real-time ultrasonography: a study in 51 patients. *Gastroenterology*, **86**, 495–502
4. Colombo, M., DeFranchis, R., Del Ninno, E., *et al.* (1991) Hepatocellular carcinoma in Italian patients with cirrhosis. *N.Engl.J.Med.*, **325**, 675–680
5. Okuda, K., Fujimoto, I., Hanai, A. and Urano, Y. (1987) Changing incidence of hepatocellular carcinoma in Japan. *Cancer Res.*, **47**, 4967–4972
6. Colombo, M., Kuo, G., Choo, Q.L. *et al.* (1989) Prevalence of antibodies to hepatitis C virus in Italian patients with hepatocellular carcinoma. *Lancet*, **2**, 1006–1008
7. Okuda, K. (1992) Hepatocellular carcinoma: recent progress. *Hepatology*, **15**, 948-963
8. Edmondson, H.A. and Steiner, P.E. (1954) Primary carcinoma of the liver: a study of 100 cases among 48,900 necropsies. *Cancer*, **7**, 462–503
9. Ebara, M., Ohto, M., Shinagawa, T. *et al.* (1986) Natural history of minute hepatocellular carcinoma smaller than three centimeters complicating cirrhosis. *Gastroenterology*, **90**, 289–298
10. Calvet, X., Bruix, J., Gines, P. *et al.* (1990) Prognostic factors of hepatocellular carcinoma in the West. A multivariate analysis in 206 patients. *Hepatology*, **12**, 753–760
11. Sheu, J.C., Sung, J.L., Chen, D.S. *et al.* (1985) Growth rate of asymptomatic hepatocellular carcinoma and its clinical implications. *Gastroenterology*, **89**, 259–266
12. Nomura, F., Ohnishi, K. and Tanabe, Y. (1989) Clinical features and prognosis of hepatocellular carcinoma with reference to serum alpha-fetoprotein levels. Analysis of 606 patients. *Cancer*, **64**, 1700–1707

Kunio Okuda, MD, PhD.
First Department of Medicine
Chiba University School of Medicine
Chiba 260
Japan

### BUDD-CHIARI SYNDROME: SHUNT OR TRANSPLANT?

### ABSTRACT

*Shaked, A., Goldstein, R.M., Klintmalm, G.B., Drazan, K., Husberg, B. and Busuttil, R.W. (1992) Portosystemic shunt versus orthotopic liver transplantation for the Budd-Chiari Syndrome. Surgery, Gynecology & Obstetrics; 174: 453–459.*

**We have analyzed the indications and results of shunt operation versus orthotopic liver transplantation (OLT) in 22 patients with Budd-Chiari syndrome (BCS). The underlying cause of the syndrome was similar between the two groups and was related to myeloproliferative disorders or the use of birth control pills in 18 of 22 patients. The results of biopsies of the liver showed centrilobular congestion and necrosis in all candidates who underwent shunting and the presence of fibrosis and cirrhosis in the OLT candidates. The indications for shunts included symptoms related to portal hypertension only and well-preserved synthetic hepatic function. Ten patients were treated with 12 shunt procedures, including mesoatrial (eight patients) and side to side portacaval shunt (four patients). Significant complications**

